# Mechanical stimulation of human hair follicle outer root sheath cultures activates adjacent sensory neurons

**DOI:** 10.1126/sciadv.adh3273

**Published:** 2023-10-27

**Authors:** Julià Agramunt, Brenna Parke, Sergio Mena, Victor Ubels, Francisco Jimenez, Greg Williams, Anna DY Rhodes, Summik Limbu, Melissa Hexter, Leigh Knight, Parastoo Hashemi, Andriy S. Kozlov, Claire A. Higgins

**Affiliations:** ^1^ Department of Bioengineering, Imperial College London, London, UK.; ^2^ Mediteknia Clinic, Las Palmas, Gran Canaria, Spain.; ^3^ University Fernando Pessoa Canarias, Gran Canaria, Spain.; ^4^ Farjo Hair Institute, London, UK.; ^5^ Procter & Gamble, Reading, UK.

## Abstract

Mechanical stimuli, such as stroking or pressing on the skin, activate mechanoreceptors transmitting information to the sensory nervous system and brain. It is well accepted that deflection of the hair fiber that occurs with a light breeze or touch directly activates the sensory neurons surrounding the hair follicle, facilitating transmission of mechanical information. Here, we hypothesized that hair follicle outer root sheath cells act as transducers of mechanical stimuli to sensory neurons surrounding the hair follicle. Using electrochemical analysis on human hair follicle preparations in vitro, we were able to show that outer root sheath cells release ATP and the neurotransmitters serotonin and histamine in response to mechanical stimulation. Using calcium imaging combined with pharmacology in a coculture of outer root sheath cells with sensory neurons, we found that the release of these three molecules from hair follicle cells leads to activation of sensory neurons.

## Teaser

Electrochemical analysis reveals that epithelial cells from human hair follicles can release serotonin and histamine.

## INTRODUCTION

The hair follicle is a complex dynamic structure that is present in the skin of all mammals. Hair follicles have many functions, such as thermoregulation, sweat production, and skin protection (*1*). In rodents and other mammals, vibrissa (whisker) follicles have known roles in mechanoprocessing (*2*). Humans lack whisker follicles and have instead developed our sense of touch, which enables both physical (navigation and perception) and emotional (pleasure and neurodevelopment) responses.

Low-threshold mechanoreceptors (LTMRs) are sensory neurons present within the skin that transfer tactile information to the brain. In glabrous skin (nonhairy skin located on the palms of our hands and soles of our feet), this transfer facilitates an understanding of our external surroundings (*3*, *4*). Hairy skin also contains C-LTMRs, a specific type of tactile neuron that is not present on glabrous sites and is involved in processing affective touch response (*5*, *6*). Various mechanical stimuli, such as stretch, skin movement, compression, or hair fiber deflection can activate these different classes of mechanoreceptors within the skin (*4*, *7*). While mechanoreceptors were originally assumed to be located on the sensory neurons, recent data have shown how Merkel cells and keratinocytes, both located in the skin epidermis, play a role in touch perception and can also be classified as mechanoreceptors (*8*–*10*). While Merkel cells release noradrenaline and serotonin in response to tactile stimuli, keratinocytes release adenosine 5′-triphosphate (ATP). In turn, these molecules activate sensory neuron afferents associated with the Merkel cell complex and skin epidermis, respectively (*8*, *11*, *12*).

After activation of mechanoreceptors, sensory nerve endings within the skin are then involved in sending tactile information to the brain. In mouse skin, the physiology and morphology of these nerve endings have been described in detail, revealing that in addition to the skin epidermis, hair follicles are innervated by a heterogeneous group of sensory neurons with longitudinal and circumferential morphology (*4*, *13*, *14*). While several groups have shown the presence of axons around human hair follicles, a clear picture of their molecular identity as well as their longitudinal and circumferential end-organ morphology has not been described (*15*–*18*).

Despite the complex innervation patterns localized around the hair follicle in hairy skin, mechanotransduction capacity has not been attributed to cells within the hair follicle. We hypothesized that hair follicle epithelial cells, which we shall refer to hereon as outer root sheath (ORS) cells, act as a transducer when the hair fiber is deflected, facilitating the activation of sensory neuron afferents that wrap around the follicle. In this work, we used a combination of immunolabeling, calcium imaging, and voltammetry on in vitro and ex vivo preparations of human hair follicles to investigate this system’s role in processing tactile information.

## RESULTS

### Humans have morphologically and molecularly distinct mechanoreceptors innervating terminal hair follicles

**Fig. 1.**
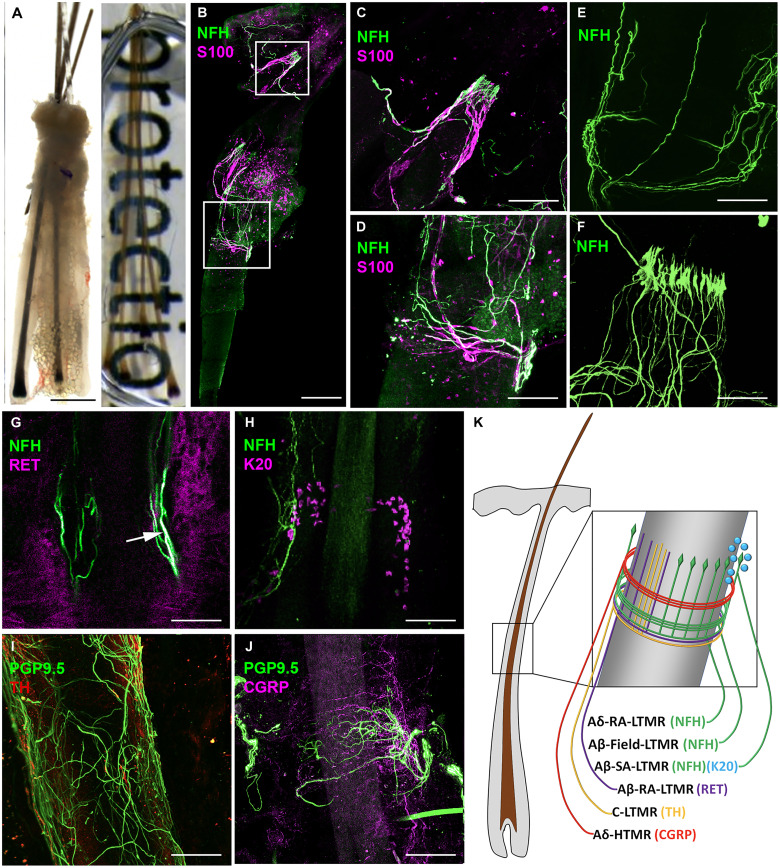
Human occipital scalp hair follicles are innervated by several LTMRs and an HTMR. (**A**) Example of a human follicular unit from scalp skin composed of terminal hairs before (left) and after (right) clearing process. (**B**) Follicular unit stained with NFH and S100 showing longitudinal (**C**) and circumferential (**D**) nerve endings from Aδ-RA-LTMR, Aβ-RA-LTMR, and Aβ-Field-LTMR. (**E**) Volumetric stacking of human follicular units showing LTMR circumferential endings. (**F**) Whole-mount images created using volumetric stacking of human follicular units and 3D rendering show the lanceolate shape of LTMR longitudinal endings. (**G**) Overlap (arrow) of RET and NFH antibody staining reveals Aβ-RA-LTMR longitudinal nerve ending. (**H**) NFH antibody reveals Aβ-SA-LTMR innervating K20^+^ Merkel cells. (**I**) Antibody against TH identifies C-LTMR longitudinal endings (**J**) Calcitonin gene-related peptide (CGRP) antibody identifies Aδ-HTMR forming circumferential endings around human hair follicles. (**K**) Schematic representation of the different LTMRs and high-threshold mechanoreceptor (HTMR) that can innervate human hair follicles. While depicted on the schematic for visual clarity, we do not know if these nerves endings are all present on every terminal hair follicle or if there is heterogeneity between follicles in terms of their innervation. Scale bars, (A) 500 μm, (B) 200 μm, (B, i) 100 μm, and [B (ii) and C to H] 50 μm.

In human scalp, hair follicles grow in follicular units with three to four hairs per unit. Unlike mouse skin, where there are several different types of follicles, in human skin, hair follicles are classified as either terminal or vellus. To assess the architectural arrangement of circumferential and longitudinal nerve endings (*19*) around terminal human hair follicles, we performed whole-mount immunostaining with volumetric and three-dimensional (3D) imaging in cleared human follicular units taken from occipital scalp skin (Fig. 1A and fig. S1). First, to investigate LTMR innervation, we used an antibody against the neuronal marker neurofilament heavy (NFH) chain, which is expressed in circumferential and longitudinal large and medium diameter LTMRs (*20*), combined with an S100 antibody, which binds to myelin-forming Schwann cells (Fig. 1B) (*21*). We found that human follicular units are innervated by NFH^+^ LTMR subtypes, with localization around and above the K15^+^ region of the hair follicle that contains hair follicle stem cells (fig. S1). These NFH^+^ neurons formed both circumferential (Fig. 1C) and longitudinal (Fig. 1D) endings and a proportion had S100^+^ myelinated afferents. Consequently, these neurons can be classified as either longitudinal Aδ-RA-LTMR, longitudinal Aβ-RA-LTMR, or circumferential Aβ-Field-LTMR, a newly found LTMR that is responsive to skin stroking (*22*). To better assess the structure and shape of the NFH^+^ nerve endings, we used high-resolution confocal microscopy with 3D rendering and identified circumferential (Fig. 1E) and lanceolate (Fig. 1F) structures in a collar around the upper permanent region of the hair follicle. With NFH^+^ staining, it is not possible to distinguish between longitudinal Aδ-RA-LTMR and longitudinal Aβ-RA-LTMR. Therefore, to specifically assess whether Aβ-RA-LTMR were present around human hair follicles, we used an antibody against RET receptor, which marks Aβ-RA-LTMR, alongside NFH. We found that these antibodies labeled neurons that form longitudinal nerve endings around human hair follicles, which match the features of Aβ-RA-LTMR found in mice (*4*) (Fig. 1G).

To further investigate the morphology of other tactile receptors around human hair follicles, we expanded our whole-mount immunostaining analysis to include more antibodies. Using a combination of K20 and NFH antibodies, we found NFH^+^ nerve endings innervating K20^+^ Merkel cells (Fig. 1H). A direct ORS-neural connection was also visible with NFH innervation in regions devoid of K20^+^ cells (fig. S2). We found that an antibody against tyrosine hydroxylase (TH), which marks slowly adapting C-LTMRs, labeled neurons forming longitudinal nerve endings (Fig. 1I). Dual staining of TH [which also stains the sympathetic nerve in the arrector pili muscle (APM)] and NFH highlights the localization of LTMR above the APM insertion point (fig. S3). Last, using an antibody against calcitonin gene-related peptide (CGRP), which labels high-threshold mechanoreceptors (HTMRs) (*23*), we found positive staining in circumferential nerve endings of human hair follicles (Fig. 1J). Here, using a collective of antibodies, we have identified the presence of longitudinal Aδ-RA-LTMR and Aβ-RA-LTMR, circumferential Aβ-Field-LTMR, Aβ-SA-LTMR, longitudinal C-LTMR, and circumferential Aδ-HTMR around human scalp hair follicles (Fig. 1K). Because of cross reactivity of antibodies with one another, we were unable to investigate whether there was heterogeneity in terms of innervation patterns between follicles.

### ORS cells from human hair follicles have mechanosensitive properties

**Fig. 2.**
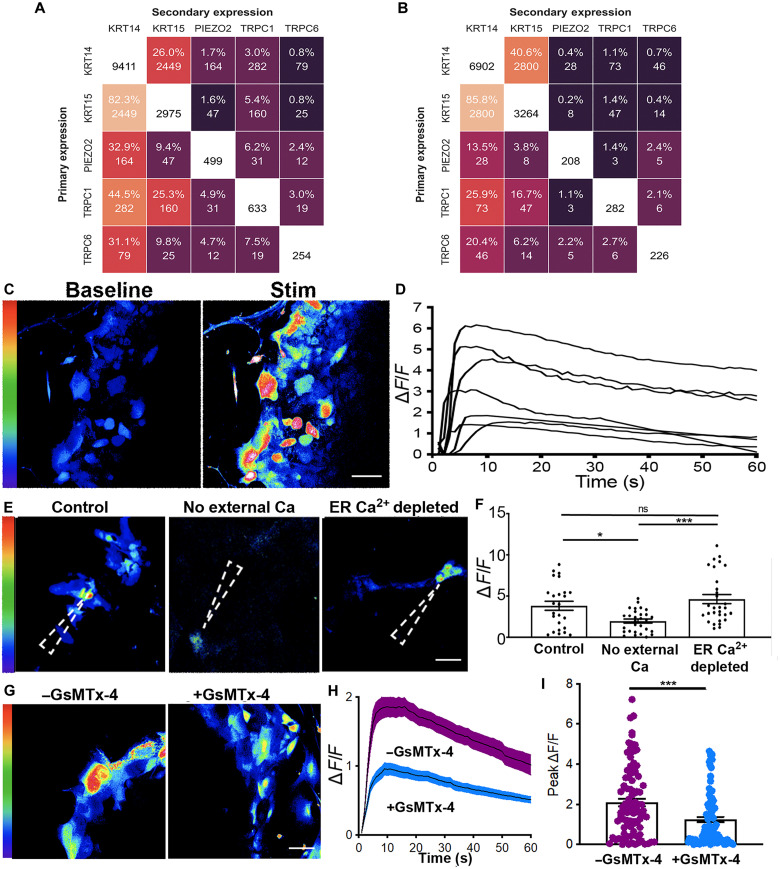
ORS cells in vitro respond to mechanical stimulus in vitro. (**A**) Heatmap depicted coexpression of scRNA-seq data of human scalp hair follicles showing that 5.4% of K15 cells also express TRPC1 (**B**) Coexpression analysis of scRNA-seq of human skin showing that 1.1% of K14-expressing cells also express TRPC1 (**C**) Representative images of ORS cells before (baseline) and after they were mechanically activated (stim). (**D**) Δ*F*/*F* traces of mechanically activated calcium transients in ORS cells from (C). (**E**) Representative images of ORS cells after mechanical activation under +Ca^2+^ −TPSG (control), −Ca^2+^ −TPSG (no external Ca^2+^), and +Ca^2+^ +TPSG (ER Ca^2+^ depleted) conditions. The dashed arrowhead shows the point of stimulus. (**F**) Graph showing peak Δ*F*/*F* from *n* = 2 ORS cultures from two patients (total of 25 to 30 cells in each condition). A one-way analysis of variance (ANOVA) with a Bonferroni post hoc test was performed to compare the peak values of Δ*F*/*F* from all stimulated cells across the three conditions. (**G**) Representative images of ORS cells after stimulation with and without GsMTx-4. (**H**) Mean Δ*F*/*F* traces of mechanically stimulated calcium transients in ORS cells with and without GsMTx-4. (**I**) Graph showing Δ*F*/*F* from *n* = 7 ORS cultures from four patients (total of 90 cells in the –GsMTx-4 group and 90 in the +GsMTx-4 group). An unpaired *t* test was used to compare the Δ*F*/*F* values in cells within each condition. Scale bars, (C and G) 20 μm and (E) 50 μm. *P < 0.05 and ***P < 0.001. ns, not significant.

After showing that human scalp hair follicles are innervated by different types of LTMRs and an HTMR, we next investigated whether ORS cells have mechanosensory properties that can modulate the LTMR response, as observed in the Merkel cell neurite complex (*11*). First, we reanalyzed a published single-cell RNA sequencing (scRNA-seq) dataset of 19,083 cells (after quality control) isolated from human hair follicles to assess the presence of cells with mechanotransductive potential. While our polymerase chain reaction (PCR) data and immunostaining show the presence of K20^+^ cells in the human hair follicle (fig. S4), we did not find any *K20*- or *ATOH1*-positive cells within the dataset, indicating that no Merkel cells were captured during the single-cell preparation protocol and sequencing. Instead, we found that several cell clusters expressed the mechanotransductive ion channels *PIEZO1*, *PIEZO2*, *TRPC1*, and *TRPC6* (fig. S5) (*24*). When we looked specifically at the ORS cell cluster, we found that the fold change of these genes was relatively low. As this is a common occurrence in complex nonhomogenous populations, we decided to look at coexpression within individual cells rather than cell clusters. After performing quality control with CO-expression Tables ANalysis (COTAN), we were left with 18,832 cells within the hair follicle dataset that were analyzed for coexpression. Using COTAN, we found that 282 (3%) of the 9411 *K14*-expressing cells and 160 (5.4%) of the 2975 *K15*-expressing cells also expressed *TRPC1*. For *PIEZO2*, 1.7% of *K14*-expressing cells and 1.6% of *K15*-expressing cells also expressed this ion channel in the hair follicle scRNA-seq dataset (Fig. 2A).

Next, to compare mechanosensitive machinery in hair with skin, we analyzed a published scRNA-seq dataset of 23,813 cells (after quality control) isolated from human skin and again looked for coexpression of mechanosensitive ion channels with epithelial cells. Here, we found that of the 6902 *K14*-expressing cells, just 28 (0.4%) expressed *PIEZO2*, while 73 (1.1%) also expressed *TRPC1* (Fig. 2B). These data point to the human hair follicle having enhanced (approximately threefold higher) mechanosensitive machinery relative to the skin.

Next, to functionally analyze if cells from the hair follicle can respond to mechanical stimuli, we used an explant approach to obtain cultures of ORS cells that we could use for testing. Note that *K20*, *VIL1*, and *ATOH1* were not expressed in these cells, indicating that Merkel cells are not present within the ORS cultures (fig. S4). In mechanosensory cells, an applied tension to the cell membrane opens mechanosensitive ion channels, which permits the influx of calcium from the extracellular space, increasing the calcium concentration in the cytoplasm (*25*, *26*). To understand if ORS cells in vitro can respond to a mechanical stimulus, we incubated them with the fluorescent calcium indicator Cal-520, which allows for quantification of changes in intracellular calcium concentrations (*27*). After obtaining baseline recordings, we selected and stimulated ORS cells using a 5-μm mechanical displacement, then evaluated the change (Δ*F*/*F*) in intracellular calcium within each stimulated cell (Fig. 2C). We found that the mechanical displacement elicited an increase in intracellular calcium concentration, which was sustained for at least 60 s in some instances, suggesting that ORS cells in vitro do respond to mechanical stimulus (Fig. 2D). There was also extensive variability in the intensity and duration of the cellular response in ORS cells, although most of the cells were responsive.

To determine the source of the calcium that mediates the increase of Δ*F*/*F*, we incubated cells with thapsigargin (TPSG), an inhibitor of sarco/endoplasmic reticulum Ca^2+^ adenosine triphosphatase (ATPase) (SERCA), thereby depleting ER Ca^2+^ stores. Cells preloaded with Cal-520 in three different conditions—+Ca^2+^ −TPSG (control), −Ca^2+^ −TPSG (extracellular Ca^2+^ removed), and +Ca^2+^ +TPSG (ER Ca^2+^ depleted)—were then stimulated as described above (Fig. 2E). The intracellular calcium concentration increase after mechanical stimulation was significantly smaller in the absence of extracellular Ca^2+^ compared to the controls (Fig. 2F and table S1). While the calcium concentration in TPSG-treated cells after mechanical stimulation was higher than in the control, this difference was not statistically significant (Fig. 2F). These results indicate that the increase in calcium concentration in ORS cells after mechanical stimulation is mainly due to Ca^2+^ influx from the extracellular space.

Next, to evaluate if the calcium response after mechanical stimulation is mediated by the activation of mechanosensitive ion channels such as Piezo and Transient Receptor Potential (TRP) channels, we treated ORS cells with GsMTx-4, a mechanosensitive and stretch-activated ion channel inhibitor (Fig. 2, G and H). We observed that the Δ*F*/*F* calcium peak within ORS cells is approximately twofold lower after incubation with GsMTx-4 compared to when no GsMTx-4 was used (Fig. 2I). This result suggests that mechanosensitive ion channels are present in the membranes of ORS cells where they can be activated by mechanical forces, such as those originating during hair shaft deflection.

### Mechanically activated ORS cells activate sensory neurons

After confirming that ORS cells respond to mechanical stimulus, we assessed their ability to transmit this signal to associated LTMRs. We cocultured human ORS cells with LTMRs isolated from murine dorsal root ganglia (DRGs). From the DRGs, we obtained a heterogeneous culture of neurons including NFH^+^ and PGP9.5^+^ Aδ-LTMRs, Aβ-LTMRs, and TH^+^ C-LTMRs (fig. S6). Within the cocultures, we observed DRGs and ORS cells in close proximity to one another (fig. S7), suggesting that this in vitro model is suitable to investigate signaling between these two cell types.

**Fig. 3.**
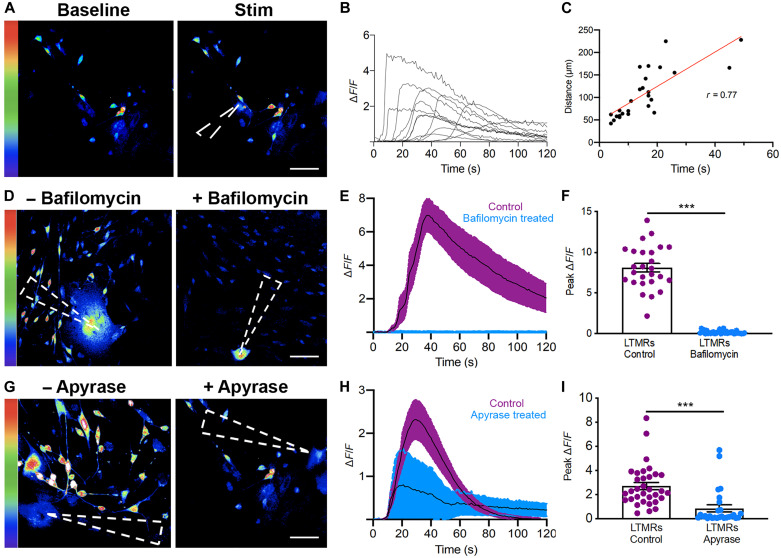
Mechanically stimulated ORS cells evoke calcium transients in LTMRs. (**A**) Images showing LTMR calcium transients before and after ORS cell activation (baseline and stim, respectively). (**B**) Representative traces of Δ*F*/*F* in LTMRs after mechanical activation of ORS cells. (**C**) Correlation of time to calcium transient in LTMR with distance from activated ORS cell. (**D**) Images of LTMRs with or without bafilomycin after ORS cell activation. (**E**) Mean Δ*F*/*F* in LTMRs with or without bafilomycin after ORS stimulation. (**F**) Graph showing peak Δ*F*/*F* in *n* = 2 LTMR cultures from one patient (26 different cells under each condition). An unpaired *t* test was used to compare the Δ*F*/*F* in all cells between the control and drug-treated conditions. (**G**) Images of LTMRs with or without apyrase after ORS cell stimulation. (**H**) Mean Δ*F*/*F* in LTMRs with or without apyrase after ORS activation. (**I**) Graph showing peak Δ*F*/*F* in *n* = 4 LTMR cultures from three patients (total 33 and 27 cells under each condition, respectively). An unpaired *t* test was used to compare the peak Δ*F*/*F* in all cells from each condition. The dashed arrowheads in (A), (D), and (G) show the point of stimulus. Scale bars, (A, D, and G) 50 μm. ***P < 0.001

We next used the ORS-LTMR cocultures to evaluate if there was an excitatory connection between ORS cells and associated LTMRs. To assess LTMR activation, we again looked at the levels of intracellular calcium (in cocultures incubated with Cal-520), but this time within the LTMRs as opposed to the ORS cells. ORS cells were selected and stimulated as described previously (Fig. 3A). We found that within this culture system, neurons located 70 to 200 μm from the point of stimulation increased their intracellular calcium levels in response to ORS cell stimulation (Fig. 3B and movie S1). LTMR intracellular calcium started increasing from approximately 10 s after ORS mechanical stimulation; however, in some LTMR, the activation took more than 50 s from ORS stimulation. Comparison of the time to activation with the distance from the point of ORS stimulation revealed a strong correlation (*R* = 0.77), suggesting that molecules released from the ORS diffuse through the media to activate neurons (Fig. 3C). It should be reiterated that this in vitro ORS-LTMR coculture, where molecules can diffuse through the media, is quite different from the follicle-neural connections present in vivo.

To confirm if a signaling molecule released from ORS cells was mediating neuronal response, we evaluated LTMR response in cocultures incubated for 24 hours with the antibiotic bafilomycin A1, a vacuolar-type H^+^-ATPase inhibitor. This procedure aims at dissipating the vesicular proton gradient, which is necessary for vesicular transporters to pump transmitter molecules into the vesicles, thus resulting in the eventual depletion of synaptic vesicles. We mechanically stimulated ORS cells in cocultures preloaded with Cal-520 as described previously and measured the calcium concentration in both ORS cells and LTMRs (Fig. 3D). While ORS cell calcium transients were not significantly affected by bafilomycin (fig. S8A), the LTMR response was completely abrogated (Fig. 3, E and F). When we directly stimulated the LTMRs incubated with bafilomycin, their ability to increase intracellular calcium levels was, as expected, unaffected (fig. S8B).

Because ATP is a known signaling molecule between skin keratinocytes and sensory neurons (*28*), we decided to evaluate its role in ORS-LTMR communication. We incubated our coculture system with apyrase, an ATP diphosphohydrolase that catalyzes the conversion of ATP to adenosine 5′-diphosphate (ADP) but does not affect sensory neuron excitability (*8*). When we delivered a mechanical stimulus to ORS cells as previously described, we found that apyrase incubation abrogated the calcium response in 70.3% of nearby LTMRs (Fig. 3, G and I). Conversely, 29.7% LTMRs in the apyrase-treated cultures still responded to ORS stimulation by increasing their intracellular calcium, revealing that ORS cells do not exclusively use ATP for communication with LTMRs. This result implicates additional signaling pathways in the cross-talk between ORS cells and sensory neurons.

### ORS cells from human hair follicles release serotonin and histamine upon mechanical stimulation

**Fig. 4.**
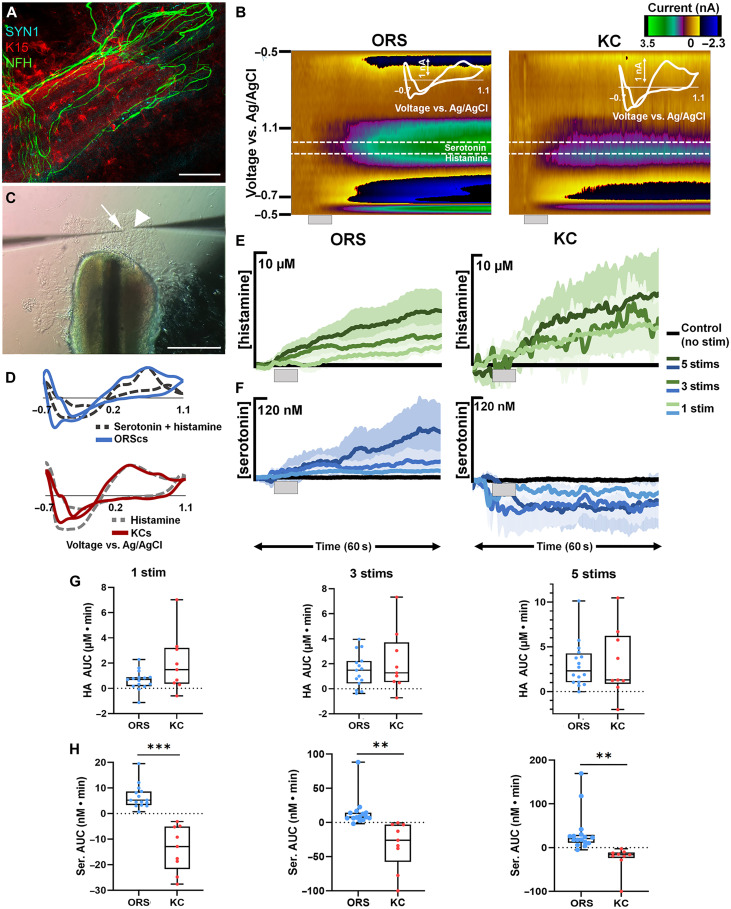
FSCV analysis of neurochemical release in epithelial cell types. (**A**) Whole-mount immunostaining of cleared human follicular units using SYN1, NFH, and K15 antibodies. (**B**) Representative color plots of stimulated release in ORS cells and keratinocytes (KC) with inset cyclic voltammograms (CVs). Gray bar denotes the time and duration of the stimulation. (**C**) Image of an ORS cell explant culture with a CFM (arrow) and stim pipette (arrowhead). (**D**) Normalized CVs from the ORS cells (blue) and a mixed flow injection analysis (FIA) injection of 150 nM serotonin and 5 μM histamine (dark gray dashes) and from the keratinocytes (red) and a FIA injection of histamine (light gray dashes). (**E**) Average traces of stimulated release of histamine in the ORS cells and keratinocytes from one to five stims (light to dark green). (**F**) Average traces of stimulated release of serotonin in the ORS cells and keratinocytes from one to five stims (light to dark blue). (**G**) Box and whisker plots comparing the area under the current versus time curves of histamine (HA) release from the ORS cells (blue) to keratinocytes (red) from one to five stims (left to right). (**H**) Box and whisker plots comparing the area under the current versus time curves of serotonin (Ser) release from the ORS cells (blue) to keratinocytes (red) from one to five stims (left to right). *n* = 15 stimulations in five cultures from four patients for ORS cells, while in KC, *n* = 9 stimulations in three cultures from one patient. **P < 0.01 and ***P < 0.001.

Because ORS cells only partially use ATP to transmit mechanical stimuli to LTMRs, we explored other signaling molecules potentially involved in ORS-LTMR communication and investigated whether the ORS cells had the cellular machinery to release neurotransmitters. The potent effect of bafilomycin (Fig. 3F) indicated vesicular neurotransmitter release by ORS cells. We therefore performed whole-mount immunostaining of cleared human follicular units using an antibody for Synapsin I (SYN1), expressed on the surface of synaptic vesicles, and found the presence of SYN1^+^ synaptic vesicles between lanceolate nerve endings from NFH^+^ LTMRs and K15^+^ ORS cells (Fig. 4A and fig. S9). The expression of SYN1 in hair follicles reinforced our conclusion that neurotransmitters mediate communication between ORS cells and LTMRs. Our next step, therefore, was to identify these neurotransmitters.

To identify and quantify neurochemicals released from these ORS cells, we used fast scan cyclic voltammetry (FSCV) at surface-modified carbon fiber microelectrodes (CFMs) in ORS explant cultures (Fig. 4, B and D). FSCV is an electrochemical method that uses analyte-optimized voltage schemes (waveforms) applied at fast scan rates (100 to 1000 s V s^−1^) to oxidize and reduce electroactive species at distinct potentials. Here, we used a waveform optimized to accurately detect electrochemical signatures of multiple analytes simultaneously. We chose this waveform because it is used in vivo to codetect serotonin and histamine release (*29*). In this study, we were initially unsure of which neurochemicals would be released from these cells and wanted to be able to capture the oxidation of multiple analytes at their unique oxidation potentials in one measurement.

In the explant cultures, we placed the CFM 1 to 3 μm above the cells and the stimulation electrode (stim) near the CFM. We used the 5-μm displacement stimulation protocol used throughout this study one, three, or five times (1 s apart) to assess whether the total number of mechanical stimuli affected the cellular release profile. Upon stimulation, we found the ORS cells to corelease serotonin and histamine, shown by a representative color plot (Fig. 4B) The signal was confirmed to be serotonin and histamine by normalizing a cyclic voltammogram (CV) of 5 μM histamine and 150 nM serotonin taken in a flow injection analysis (FIA) system to a CV from the ORS cell culture, which showed overlapping oxidative peaks (Fig. 4D). The FSCV signals were then converted to concentration via post-calibration curves collected in a FIA system for each identified species alone and for their binary mixtures. Upon stimulation, the current versus time (*I-T*) traces showed a proportional release to increasing number of stimulations, for both serotonin and histamine (Fig. 4, E and F), with AUC analysis quantifying this for histamine (one stim: 0.65 ± 0.20 μM∙min; three stims: 1.47 ± 0.35 μM∙min; five stims: 2.96 ± 0.63 μM∙min) and serotonin (one stim: 6.63 ± 1.20 nM∙min; three stims: 14.47 ± 5.50 nM∙min; five stims: 33.82 ± 11.32 nM∙min). These data show that ORS cells release both serotonin and histamine in response to mechanical stimulation and that the magnitude released is proportional to the number of mechanical stimuli applied. To confirm that bafilomycin was indeed eliciting its effect in ORS cells by perturbing vesicular neurotransmitter release, we used FSCV to assess ORS cells before and after an acute treatment with bafilomycin. In response to mechanical stimulation, cells treated with bafilomycin released lower levels of both serotonin (*P* = 0.0048) and histamine (*P* = 0.0504) (fig. S10).

We next investigated whether the proportional response of ORS cells to increasing number of stimuli was a specific characteristic of hair follicle epithelial cells in vitro or common to skin epithelia in vitro by applying the same stimulation protocol, described above, to explant cultures of interfollicular skin keratinocytes. We applied the same waveform used in ORS cultures to probe these keratinocyte cultures. As shown in the representative color plot (Fig. 4B) and verified by normalizing a CV of histamine to a CV from the keratinocyte culture, keratinocytes robustly responded to stimulation by releasing histamine (Fig. 4D). AUC analysis showed that histamine release was proportional to the number of stimuli (one stim: 1.96 ± 0.77 μM∙min; three stims 2.13 ± 0.82 μM∙min; five stims: 3.20 ± 1.28 μM∙min) in a similar manner to the ORS cells (Fig. 4E). Unlike the ORS cells, the keratinocytes did not release serotonin in response to the stimulation (Fig. 4F), quantitated by AUC analysis (one stim: −13.75 ± 2.97 nM∙min; three stims: −33.55 ± 12.25 nM∙min; five stims: −26.50 ± 11.37 nM∙min). The apparent drop in signal after stimulation is likely an artifact of the stimulation (which can be observed in the absence of release). These results not only support previous reports of pharmacologically stimulated histamine release in keratinocytes but also show that both hair follicle and skin epithelial cells proportionally respond to mechanical stimulation (Fig. 4G). Moreover, they demonstrate that serotonin release in response to mechanical stimulation is a hallmark of hair follicle ORS cells (Fig. 4H).

### Serotonin and histamine from hair follicle ORS can modulate LTMR response

After finding that ORS cells can release both serotonin and histamine in response to tactile stimulus, we next assessed if this neurotransmitter release from ORS cells can modulate LTMR response. For this, the previously described coculture system between ORS cells and LTMRs was re-established; however, in this current iteration, cocultures were treated with mirtazapine, a serotonin (5HT2 and 5HT3) and histamine (H1) receptor antagonist.

**Fig. 5.**
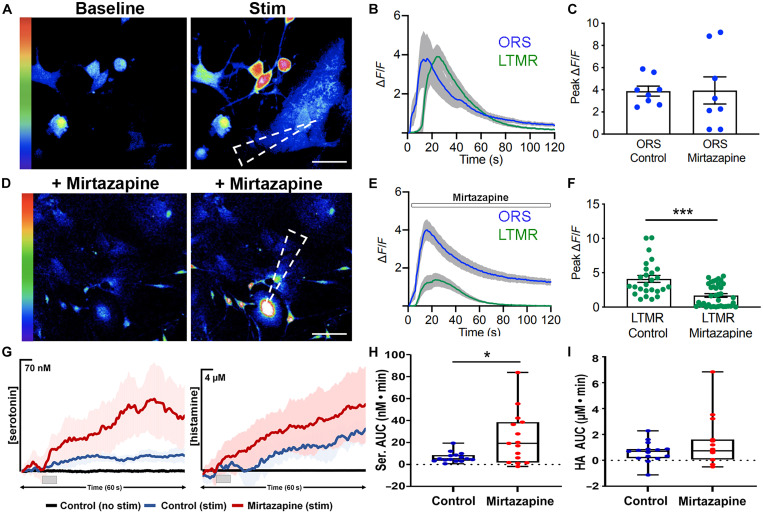
Serotonin and histamine released from ORS cells can modulate LTMR response. (**A**) Images of baseline and stimulated calcium transients in ORS cells and associated LTMRs in coculture. (**B**) Traces of Δ*F*/*F* in ORS cells and LTMRs after mechanical activation of ORS cells. (**C**) Peak Δ*F*/*F* in *n* = 8 cocultures from three patients (a different ORS cell was stimulated in each coculture). An unpaired *t* test was used to compare the peak Δ*F*/*F* values in control cells and after the drug treatment. (**D**) Images of baseline and stimulated calcium transients in ORS cells and associated LTMRs in cocultures treated with mirtazapine. (**E**) Traces of Δ*F*/*F* in ORS cells and LTMRs treated with mirtazapine after mechanical activation of ORS cells. (**F**) Peak Δ*F*/*F* in *n* = 8 cocultures from three patients (total 26 or 36 LTMRs). An unpaired *t* test was used to compare the peak Δ*F*/*F* values of the LTMRs in control and after the drug treatment (different cells within the same culture dish were used under the two conditions). (**G**) Average traces of stimulated release of serotonin (left) and histamine (right) from ORS cells with one stim. (**H**) Box and whisker plots comparing the area under the current versus time curves of serotonin release from the ORS cells from one stim. (**I**) Box and whisker plots comparing the area under the current versus time curves of histamine release from the ORS cells from one stim. *n* = 15 stimulations in five ORS cultures from four patients. The dashed arrowheads in (A) and (D) show the point of stimulus. Scale bars, (A) 20 μm and (D) 50 μm. *P < 0.05 and ***P < 0.001

After preincubation with Cal-520, baseline and evoked calcium transients were recorded in both ORS cells and LTMRs (Fig. 5, A and B). Cocultures were next incubated with mirtazapine for 30 min, and baseline and evoked calcium transients in response to 5-μm displacement were recorded (Fig. 5, C to F). While mirtazapine had no effect on calcium transients in ORS cells (*P* = 0.967) (Fig. 5C) the peak calcium recorded in LTMRs was decreased by 2.42-fold after drug treatment (Fig. 5F). The ability of LTMRs to increase their intracellular calcium was unaffected, as direct stimulation of LTMR elicited a normal response in these cells (fig. S11).

Last, to confirm that the reduced LTMR response is due to the antagonistic effect of mirtazapine on serotonin and histamine receptors and not a consequence of impaired serotonin and histamine release from ORS cells treated with mirtazapine (an off-target effect), we performed FSCV with ORS cells after mirtazapine incubation (Fig. 5G). We found that mirtazapine treatment resulted in a 3.6-fold increase in serotonin (*P* = 0.010) and a 2.1-fold increase in histamine (*P* = 0.17) release, respectively with one stim (Fig. 5, H and I, and fig. S12). This result highlights that in the presence of more serotonin and histamine (after mirtazapine incubation of ORS cells), the LTMR response still halved. Overall, these results suggest that ORS cells in human hair follicles can release serotonin and histamine in response to tactile stimulus, which, in turn, can modulate LTMR intracellular calcium concentrations through 5HT2, 5HT3, and H1 receptor activation.

## DISCUSSION

In this body of work, we set out to evaluate our hypothesis that hair follicle ORS cells can act as a signal transducer for mechanical stimulus, leading to excitation of sensory neurons surrounding the hair follicle. Using whole-mount volumetric imaging of human hair follicles, we showed follicular innervation by five different types of LTMRs and one HTMR. To understand how the follicle can interact with these LTMRs, we evaluated the mechanosensitive properties of cultured ORS cells and found that they release both histamine and serotonin in response to mechanical stimulation in a stimulus-dependent manner. This release is likely vesicular: It was calcium dependent, it was blocked by bafilomycin, and a synaptic vesicle marker SYN1 was detected in ORS cells. In contrast, interfollicular skin keratinocytes release comparable levels of histamine but only negligible levels of serotonin in response to the same stimulation protocol. Last, we showed that blocking of serotonin and histamine receptors on LTMRs dampened their ability to respond to excitatory stimuli released from ORS cells. Collectively, these results show that the hair follicle ORS and LTMRs associated with the follicle form a neuroepithelial unit.

This differential capacity for serotonin release is an intriguing facet for discussion. ORS cells are the hair follicle equivalent of keratinocytes—signaling from the dermis during skin development leads skin keratinocytes to differentiate into hair follicle epithelium. In adult skin and in response to injury, hair follicle ORS cells can dedifferentiate into skin keratinocytes to facilitate wound closure (*30*). Despite the close association between the two epithelial cell types, we found that only hair follicle ORS released serotonin. Our finding that hair follicle ORS cells, but not skin keratinocytes, release serotonin suggests an alternate signaling pathway for mechanical stimulus that is mediated by the cells within the human hair follicle.

In addition to our main findings, we also found that ORS cells release ATP in response to mechanical stimulus and use this molecule to communicate with LTMRs. These results support numerous previous findings, which have demonstrated that mechanical stimulation of keratinocytes results in calcium-evoked ATP release (*8*). Our work builds on these previous studies, showing that hair follicle epithelial cells can also release ATP in response to tactile stimuli and that this facilitates their interaction with associated LTMRs.

Previous work has also demonstrated that both mouse and human skin keratinocytes have the enzymatic machinery to synthesize histamine (*31*). A mouse keratinocyte cell line, Pam212, also releases histamine in response to α-melanocyte stimulating hormone (*31*). Our work showing that human primary keratinocytes can release histamine in response to mechanical stimulation accords with these findings. This data may also provide clues about skin conditions such as dermatographia, which the mechanism behind remains elusive.

Last, we found that LTMRs innervate human hair follicles in a specific pattern, forming longitudinal and circumferential endings around the permanent region of the follicle. Specifically, these were around and above the insertion point for the APM, which attaches proximally to the bulge where hair follicle stem cells are located. In mice, neurons innervating the hair follicle bulge have been shown to release signals including sonic hedgehog or noradrenaline that can influence the behavior of stem cells within the follicle (*32*, *33*). Our work demonstrates that this communication occurs in the other direction as well and that the follicle cells can release neurotransmitters to convey information to the associated neuron.

A limitation of our study is that it was conducted using an in vitro model system. While this enabled accurate measurement of neurotransmitters from human cells in real time, it is worth considering the physiological relevance of the data. It has previously been shown that just three types of LTMR present within the skin respond directly to deflection of the hair fiber: Aδ-RA-LTMRs, Aβ-RA-LTMRs, and C-LTMRs (*4*). While Aβ-LTMRs and Aδ-LTMRs respond to a single deflection of the hair fiber caused by a stroke or breeze, C-LTMRs are only activated by light and slow touch and have a negligible role in processing spatial and qualitative information (*5*, *6*). Aβ-Field-LTMRs and Aβ-SA-LTMR both innervate hair follicles but unexpectedly do not respond to hair fiber deflection. While we did not observe changes in the type of analyte released in response to variations in number of stimuli, we did see a stimulus-dependent response with the concentration of analyte released increasing with an increased number of stimuli. Translating these findings to the intact skin, we conclude that ORS cells can modulate their response to touch in a stimulus-dependent manner. While there is heterogeneity in the LTMRs that innervate the follicle, we were unable to resolve if this hair follicle-neural connection is specific to or favors a particular type of LTMR. Identifying which LTMR or LTMRs the follicle can communicate with could help to explain why the ORS cells have this excitatory capacity in the first place.

Collectively, our results suggest that human hair follicle ORS cells together with LTMRs form a neuroepithelial unit, which we postulate, is involved in the detection of tactile stimuli. We have demonstrated that hair follicle ORS cells are capable of releasing ATP, histamine, and serotonin in response to tactile stimuli. We also showed that human hair follicles are innervated by various sensory neurons. The release of different signaling molecules is likely to facilitate interaction and communication with different sensory neurons surrounding the follicle, leading to a variety of physical and emotive effects within the nervous system and brain. Our work lays the foundation for future studies to evaluate how ORS cells have an excitatory capacity for specific sensory neuron activation and to assess the role of scalp hair follicles in both physical and affective touch sensation.

## MATERIALS AND METHODS

### Subjects and samples

Occipital scalp skin containing hair follicles was taken from male subjects aged 23 to 54 years undergoing hair transplant surgery at both the Mediteknia Clinic (Las Palmas de Gran Canaria) and Farjo Hair Institute (London, UK). Consent forms approved by either the Universidad de las Palmas de Gran Canaria Research Ethics Committee or the Imperial College Research Ethics Committee were used to obtain informed written consent. Keratinocyte cultures were established from skin left over after abdominoplasties, with informed written consent obtained from patients using Imperial College Research Ethics Committee–approved consent forms. All research protocols were approved by either Universidad de las Palmas de Gran Canaria Research Ethics Committee or the Imperial College Joint Research Compliance Office depending on where the samples were collected.

### Expression profiling

#### Whole-mount immunolabeling

Sample processing for volumetric imaging was performed using a modified version of iDISCO (immunolabeling enabled 3D imaging of solvent-cleared organs) technique (*34*). Freshly biopsied samples were fixed overnight at 4°C in 4% paraformaldehyde (Agar Scientific) in phosphate-buffered saline (PBS) (Gibco) and then washed in two 30-min PBS washes. Fixed samples were next dehydrated using a series of 20, 40, 60, 80, and 100% methanol/H_2_O (1 hour per wash). After methanol dehydration, samples were incubated overnight at 4°C with 66% dichloromethane/33% methanol (Sigma-Aldrich). The following day samples were washed twice with 100% methanol for 10 min then bleached with 5% H_2_O_2_ in methanol overnight at 4°C. After bleaching, samples were rehydrated in 20, 40, 60, and 80% H_2_O/methanol series followed by a 1-hour PBS wash. Last, samples were washed twice in PBS with 0.2% Triton X-100 (1 hour per wash).

After fixation and pretreatment, follicles were incubated for 12 hours at 37°C in a permeabilization solution consisting of PBS with 0.2% Triton X-100, 20% dimethyl sulfoxide (DMSO), and 0.3 M glycine. Samples were next incubated for 24 hours at 37°C in a blocking solution consisting of PBS with 0.2% Triton X-100, 6% donkey serum, 6% goat serum, and 10% DMSO. After blocking, samples were incubated with primary antibodies diluted in PBS with 0.2% of Tween 20, 0.1% of heparin solution (10 mg/ml), 5% DMSO, 3% donkey serum, and 3% goat serum (Vector Laboratories) for 24 hours at 37°C. Samples were then washed four times in PBS with 0.2% of Tween 20, 0.1% of heparin solution (10 mg/ml; 4 hours per wash) before incubation for 24 hours at room temperature (RT) with secondary antibodies diluted in PBS with 0.2% of Tween 20, 0.1% of heparin solution (10 mg/ml), 3% donkey serum, and 3% goat serum. After secondary antibody incubation, samples were washed four times (4 hours per wash) in PBS with 0.2% of Tween 20 and 0.1% of heparin solution (10 mg/ml).

After immunolabeling samples were dehydrated again with a series of 20, 40, 60, 80, and 100% methanol/H_2_O (1 hour per wash). Samples were then incubated for 3 hours at RT on a rocking platform in 66% dichloromethane/33% methanol. They were next incubated for two 10-min washes in 100% dichloromethane. Last, clearing was performed incubating the samples with 100% dibenzyl ether for 1 hour. Overall, the protocol takes 9 days to complete with the timeline optimized for whole human follicles.

Samples were mounted on a glass slide and covered with a coverslip raised 2 mm above the sample to avoid sample compression. This allowed the sample to be in a 3D contained space for volumetric imaging. Samples were imaged with Leica SP8 confocal microscope with HyD detectors to depths between 200 and 500 μm.

#### Gene expression analysis in tissue and cells

RNA was extracted from plucked hair fibers or ORS cells grown in explant culture for 2 weeks using a QIAGEN RNAeasy mini extraction kit (QIAGEN, Hilden, Germany). SuperScript IV (Thermo Fisher Scientific, MA, USA) was used to generate cDNA with oligo(dt) primers, followed by incubation in ribonuclease H. A reverse transcription PCR reaction was performed using PowerUp Sybr Green Master Mix (Thermo Fisher Scientific, MA, USA) with primers designed against Hg39 build sequences (table S2). Reactions were run on a QuantStudio 3 quantitative PCR machine for 40 cycles, and the delta delta CT (ΔΔCT) method was used to calculate expression of genes of interest relative to *GAPDH*.

### Single-cell data quality control filtering and analysis

#### Seurat

Data analysis was performed on anagen human hair follicle single-cell RNA expression data obtained from a publicly available dataset [Gene Expression Omnibus (GEO) number GSE193269] (*35*). Anagen-specific human hair follicle tissue samples are extracted for further downstream analysis. Cells that expressed >500 unique molecular identifier (UMI) counts, <6500 genes, >200 genes, and <30% mitochondrial gene expression in UMI counts were included. Doublets were systematically removed by computationally comparing cell barcodes, pooling the raw count for each gene for those samples, and dividing by the total barcodes per cell identifier such that the end number of raw counts accurately reflects the counts for that barcode. Datasets from multiple donors that account for technical differences across experimental conditions were merged using derivation from the standard integration protocol described in Seurat version 4. Seurat package, version 4.3.0 (*36*), for preprocessing and normalizing gene expression data was used. Gene expression matrices were normalized using log normalization. Subsequently, the 2000 most variably expressed genes are selected in each sample using local polynomial regression as a selection method before integration. Integration of data was achieved through anchor-based canonical correlation analysis (CCA) integration with 30 CCA dimensions to obtain the integration anchors between sample origins. To maintain complete information of gene expression outside of the high variable genes, a binary reduce function is applied to combine the elements of the given gene list and their initial values before integration, such that no gene count information is lost through the integration process.

A total of 19,083 cells remaining cells were used for graph-based clustering and visualization. First, the dataset was scaled using a conventional linear regression model. Second, dimensionality reduction was achieved by clustering cells using principal components analysis (PCA), followed by uniform manifold approximation and projection (UMAP) using the Seurat R package with 50 dimensions as suggested by the nonplateauing trend as seen in an elbow plot where the SD of the principal components is depicted. To build second-level clusters, cells belonging to subpopulations were reanalyzed separately by subsetting the relevant cluster and rerunning neighbor and cluster identification. Differentially expressed genes for each cluster were identified by Wilcoxon’s rank sum test using a return threshold of 0.01, log threshold of 0.25-fold difference, and minimum fraction detection range of 0.25. These parameters were found to show balanced results regarding the number and size of the expected clusters that may be reasonably expected within the anagen human hair follicle without redundancy following clustering. Cluster labeling was performed according to observed protein marker expression within each cluster.

#### COTAN

In addition to performing analysis of gene expression in cell clusters using Seurat, we also used COTAN (*37*), an R package built on gene pair analysis theory. Here, we analyzed both the anagen hair follicle dataset (GEO number GSE193269) (*35*) and a publicly available healthy human skin scRNA dataset (GEO number GSE147424) (*38*). With the skin dataset, we extracted only the expression matrices for the seven healthy control subjects and use this for downstream analysis. Quality control with COTAN contains an additional step on top of a standardized method (described above in the “Seurat” section) where cells with low information efficiency are removed by setting a threshold for low UMI detection efficacy cells depicted by the ν value obtained from PCA. After performing quality control with COTAN of both the GSE193269 and GSE147424, we had 18,832 cells for the human anagen hair dataset and 23,813 cells in the healthy skin dataset for analysis. Rather than comparing cell identity across datasets, a comparison is drawn using generalized contingency tables. These tables are computed for each dataset using a discrete UMI count variable *R_g_*,*_c_*. First, for each dataset a uniform population of cells is assumed to act according to a negative binomial distribution such that gene count is modeled as Poisson random variables with a rate constant following gamma distribution. Therefore, *R_g_*,*_c_* is considered to operate according to a discrete Poisson distribution and normalized such that the UMI counts may be collapsed, resulting in the binarization of expression values of each UMI into two categories {*R_g_*,*_c_* = 0} and {*R_g_*,*_c_* ≥ 1}, neglecting to consider values larger than 1 as different. Using this methodology coexpression of two genes, A and B, of interest is computed and can be categorized into coexpressed genes {*R*_*A*,*c*_ + *R*_*B*,*c*_, ≥ 1}, neither A nor B expressed genes {*R*_*A*,*c*_ + *R*_*B*,*c*_, = 0}, only A or B is expressed {*R*_*A*,*c*_ ∨ *R*_*B*,*c*_, ≥ 1}. This approach enables a direct nonbiased comparison between coexpression between datasets.

### Culture of primary cells

#### Hair follicle epithelial cells

ORS explant cultures (*39*) were established from human hair follicles taken from the occipital scalp. First, follicles were incubated in dispase for 30 min at 37°C. Forceps were then used to separate the hair follicle epithelium from the hair follicle mesenchyme. Hair fibers containing epithelial tissue were then placed on mitomycin treated NIH3T3 feeder layers in 35-mm culture dishes and left to adhere for 2 to 3 minutes. FAD medium [a 1:1 ratio of Dulbecco’s modified Eagle’s medium (DMEM) (Gibco) and Ham’s F12 medium supplemented with 10% fetal bovine serum (FBS), 2 mM l-glutamine, epidermal growth factor (10 ng/ml), insulin (5 μg/ml), 0.18 mM adenine, hydrocortisone (0.4 μg/ml), 0.1 nM cholera toxin, 2 nM triiodothyronine, and 1X penicillin-streptomycin (P/S)] was then added to cultures which were incubated at 37°C in 5% CO_2_/95% humidity for 7 to 10 days when they were used in experimental procedures.

#### Keratinocytes

Keratinocyte explant cultures were isolated human skin discarded after abdominoplasty procedures. Briefly, excess fat and dermis were removed from samples, which were then incubated overnight in dispase at 4°C. The following morning, the epidermis and dermis were manually separated with forceps. The epidermis was then chopped roughly with scissors and placed onto mitomycin treated NIH3T3 feeders. The epidermis was left to dry for 2 to 3 min to adhere to the feeders before FAD medium was added. Dishes were incubated at 37°C in 5% CO_2_/95% humidity for 7 days to facilitate outgrowths of keratinocytes from the explanted epidermis, before use in experimental procedures.

#### DRG neurons

DRG neurons were isolated from C57BL/6J mice, which were euthanized for other research purposes. The spine was removed and opened transversally, and the DRGs from C2-L5 were removed using fine forceps. DRGs were incubated with Hanks’ balanced salt solution (HBSS) solution containing collagenase II (2 mg/ml) at 37°C for 1 hour. The DRG neurons were dissociated with a fire-polished Pasteur pipette with small tip diameters and plated onto petri dishes precoated with 0.1% poly-l-lysine solution. DRG neurons were cultured with DMEM supplemented with 5% FBS and 1X P/S and incubated at 37°C in 5% CO_2_/95% humidity.

##### Coculture of DRG neurons and epithelial cells

For the coculture system, we combined mouse DRG neurons and human ORS cells. Briefly, mouse DRG neurons, isolated as described above, were seeded onto a mature ORS cell culture on NIH3T3 feeders. This coculture was maintained in FAD medium at 37°C in 5% CO2/95% humidity. Cocultures were used after observing an axonal network developing toward the ORS cells, which usually occurred after approximately 10 days.

### Calcium dynamics experiments

#### Calcium imaging and analysis

Calcium imaging was performed on ORS cultures, or ORS-LTMR cocultures grown in 35-mm dishes with glass bottoms (Ibidi, Gräfelfing, Germany) using the fluorescent calcium indicator Cal-520 (AAT Bioquest, Sunnyvale, CA, USA). Cultures were incubated with 10 μM Cal-520 in HBSS with 0.04% Pluronic F-127 for 90 min at 37°C followed by 30 min at RT. Cells were then washed once with HBSS before incubation in fresh HBSS for experimentation. Dishes were placed onto an inverted Leica SP5 confocal microscope (Leica, Wetzlar, Germany) with the temperature maintained constant at 30°C. Calcium transients were imaged once per second for 60 s using a Sentech Stc-TC33USB-AS camera (Omron Sentech, Kanagawa, Japan). Before stimulation, a 60-s baseline recoding was taken to ensure there were no fluctuations in the calcium fluorescence within cells. To stimulate, an InjectMan micromanipulator (Eppendorf, Hamburg, Germany) was used in combination with a glass pipette with a 1-μm diameter tip. The pipette was lowered slowly until it touched an ORS cell at which point-of-contact distance was recorded. The pipette was then raised, and the cell was left to rest for at least 2 min or until no activity was observed. To mechanically stimulate cells, the pipette was lowered until the point-of-contact plus 5 μm. Recordings were obtained for 120 s after mechanical stimulation. Cells (either LTMRs or ORS) were distinguished from one another by size and shape. ORS cells are flatter with well-defined border and no axonal structures, and they grow in confluent monolayers and so are present surrounded by other ORS cells. LTMRs in contrast have rounded and raised cell bodies, with axonal structures protruding from the cell body (fig. S7). We also performed post hoc staining to confirm our observations.

Calcium transients were quantified and analyzed using ImageJ. After manually selecting the cells the multiregion of interest intensity profile for a time series macro was used to quantify the calcium transients. We calculated the change in intracellular calcium concentration (Δ*F*/*F*) relative to the baseline concentration before stimulation. This was plotted as a function of time, while peak Δ*F*/*F* values were used for statistical analysis.

#### Drug interventions

To evaluate if the observed increases in calcium were due to a calcium source in the extracellular space or the ER, cells preloaded with Cal-520 were incubated at 37°C in three different conditions for 8 hours before cell stimulation: Hepes-buffered HBSS (+Ca^2+^ -TPSG), Hepes-buffered HBSS with 3 μM TPSG (+Ca^2+^ +TPSG), and modified Hepes-buffered HBSS without calcium (−Ca^2+^ −TPSG). Different cultures were used for each experiment.

To assess the role of mechanosensitive ion channels in the response of ORS cells to mechanical stimuli, GsMTx-4, a mechanosensitive and stretch-activated ion channel inhibitor was used. Cultures preloaded with Cal-520 were mechanically stimulated and calcium transients recorded. GsMTx-4, at a concentration of 8 μM in Hepes-buffered HBSS was added to cultures for 20 min at 30°C, after which time, the cells were mechanically stimulated and calcium transients were recorded.

To deplete synaptic vesicles of their neurotransmitter in ORS cells, we incubated cocultures with 10 μM bafilomycin A1 in FAD medium for 1 hour at 37°C. We then loaded cocultures with Cal-520, as described previously, before mechanical stimulation and recording of calcium transients. For FSCV, we stimulated cells with the one stim protocol as described below.

To assess the role of ATP in cell response we used apyrase, an enzyme that converts ATP to ADP and ADP to adenosine 5′-monophosphate. Cultures preloaded with Cal-520 were mechanically stimulated, and calcium transients were recorded. Apyrase, at a concentration of 200 U/ml diluted in Hepes-buffered HBSS, was added to cultures for 20 min at 30°C, after which time, the cells were mechanically stimulated and calcium transients were recorded.

Last, mirtazapine, a serotonin and histamine receptor antagonist, was used to assess LTMR response to ORS stimulation. Cultures preloaded with Cal-520 were mechanically stimulated, and calcium transients were recorded. Cultures were then incubated with 200 μM mirtazapine diluted in Hepes-buffered HBSS for 30 min at 37°C. After 30 min, different cells within the same original culture dish were mechanically stimulated and calcium transients were recorded.

### Fast scan cyclic voltammetry

#### Carbon fiber microelectrodes

This process has been previously described in detail (*40*), but, briefly, a T-650 carbon fiber (7 μm in diameter) was aspirated through a borosilicate glass capillary (Science Products GmbH, Hofheim, Germany). A carbon glass seal was made by melting and pulling the glass capillary with a vertical pipette puller (Narishige, Japan). The exposed fiber was cut to 150 μm under an optical microscope, and an electrical connection was forged from the open end of the capillary with a pinned stainless steel wire coated in silver paint. To maximize sensitivity to serotonin, the carbon surface was modified by (i) electropolymerizing a thin layer of NafionTM by applying 1 V for 30 s in a solution of NafionTM (Liquion-1105, Ion Power, New Castle, DE, USA) and then drying at 70°C for 10 min (*40*) and (ii) electropolymerizing polyglutamic acid by applying a waveform (−1.2 to 1.3 to −1.2 V at 400 V/s) at 60 Hz for 10 min in a solution of 1 μM glutamic acid in 1× tris buffer (pH 7.4) (*41*).

#### FSCV data acquisition

FSCV data were acquired using WCCV 4.0 software (Knowmad Technologies LLC, Tuscon, AZ, USA), a Dagan potentiostat (Dagan Corporation, Minneapolis, MN, USA), and a Pine Research headstage (Pine Research Instrumentation, Durham, NC, USA). A histamine-specific waveform was applied (−0.5 to −0.7 to 1.1 to −0.5 V at a scan rate of 600 V/s) at 10 Hz, as described previously (*42*). A pseudo-Ag/AgCl reference electrode was made for each experiment by electroplating chloride onto a silver wire (0.010 mm in diameter, A-M systems, Sequim, WA, USA) before each experiment.

ORS cells (five dishes from four patients) or keratinocytes (three dishes from one patient) were placed in 35-mm dishes in a solution of HBSS + 10 mM Hepes at pH 7.4. All dishes were probed in the same manner. Each dish was mounted on an inverted microscope (Olympus, Tokyo, Japan), and the CFM was placed 5 μm above the cells with a micromanipulator (Sutter Instruments, CA, USA). The voltage waveform was applied at 60 Hz for 10 min and then at 10 Hz for 10 min to equilibrate before data collection (*43*). Before stimulation, 10 control files were collected. The stimulation was applied near the CFM by indenting the cells by 5 μm with a blunted pipette tip one, three, or five times, with indents 1 s apart. A stimulation file was collected once every 10 min until each stimulation protocol was applied to the area. The CFM was moved to a different location in the dish, allowing for re-equilibration for 5 min before data collection. The process was repeated for a total of three locations in each dish, totaling 15 stimulation files for the ORS cultures and 9 stimulation files for the keratinocytes.

#### FIA experiments

After cell experiments, each CFM was post-calibrated in a flow cell with known concentrations of serotonin and histamine. All chemicals were purchased from Sigma-Aldrich (Sigma-Aldrich, St. Louis, MO, USA) unless otherwise indicated. Serotonin HCl and histamine dihydrochloride were dissolved in 1× HBSS (Thermo Fisher Scientific, Waltham, MA, USA) + 10 mM Hepes at pH 7.4 to create stock solutions. Serial dilutions were performed to create calibration solutions of serotonin (1000, 500, 100, 50, 25, and 10 nM), histamine (20, 10, 5, 2, 1, and 0.5 μM), and a mixed solution (150 nM serotonin + 5 μM histamine). FIA was performed in a custom-built flow cell (*44*), with a syringe infusion pump (Harvard Apparatus, model 70-4500, Cambridge, UK) controlling the flow at 1.7 ml/min. A pseudo-Ag/AgCl reference electrode was placed below the CFM, and the hole was plugged to seal the flow system. The CFM was mounted and lowered into the flow stream until the exposed fiber was fully submerged. The waveform was applied at 60 Hz for 10 min and then at 10 Hz for 10 min before data collection. For each data point, the analyte was introduced to the flow stream for 10 s via a six-port HPLC loop injector (Cheminert valve, VICI, Houston, TX, USA), resulting in a rectangular current versus time trace, indicative of ideal flow conditions. Each point was repeated three times per electrode.

#### FSCV data analysis

Computational models were developed and trained using Python 3.10 and the scikit-learn library. A partial least squares regression (PLSR) model for each electrode is used to convert the Faradaic current into concentration of serotonin and histamine. The calibration model was designed to reduce the dimensionality of the CV from 3000 samples (inputs) to 15 features and to simultaneously predict the concentration of serotonin and histamine (two outputs). The number of components of the regression were chosen to reduce the predictive error of in vitro flow injections used to train the model and avoid overfitting. CVs were normalized by removing the mean and scaling to unit variance (standardization). Models were trained using *k*-fold cross-validation with five different train test sets, a limit of 1000 iterations, and 1 × 10^−6^ tolerance. Flow injection signals of serotonin and histamine mixtures in HBSS buffer (see above) were used to train the model to predict these two analytes. The PLSR model had a combined coefficient of determination (*r*^2^) of 0.9803 for serotonin and histamine prediction. The root mean square errors of test predictions were 14.63 nM for serotonin and 0.69 mM for histamine. AUC of concentration versus time traces was calculated using the Simpson’s rule between time 5 s (start of stimulation) and 60 s (end of recording). The slope of the concentration release after stimulation was calculated via linear regression between time 5 and 30 s.

### Statistical analysis

Statistical significance is defined as *P* < 0.05. All statistical tests were performed using Python 3.10 SciPy library and MATLAB 2020b. Distribution of samples is shown as means ± SEM or in box whisker plots with a line at the mean value. Calcium transient data were tested for significance using a one-way analysis of variance (ANOVA) or unpaired *t* tests depending on the experimental conditions. In these experiments, each cell was regarded as a separate datapoint. Serotonin and histamine differences across the dataset were tested for significance using a two-way ANOVA and Tukey-Kramer post hoc multiple comparisons of the AUC and slope of release. In FSCV experiments, each location in the culture dish was regarded as a separate datapoint. A summary of statistical tests and *P* values can be found in table S1.
