# Supplementary material for: Mechanical stimulation of human hair follicle outer root sheath cultures activates adjacent sensory neurons
Source: Sci Adv. 2023 Oct 27;9(43):eadh3273. doi: 10.1126/sciadv.adh3273 (PMC10610912; doi:10.1126/sciadv.adh3273)
Supplement: Supplementary file 1 — Figs. S1 to S12 Tables S1 and S2 Legends for data S1 and S2 Legend for movie S1 [file sciadv.adh3273_sm.pdf]

Science Advances
AAAS

Supplementary Materials for

Mechanical stimulation of human hair follicle outer root sheath cultures activates adjacent sensory neurons

Julià Agramunt *et al.*

Corresponding author: Claire A Higgins, c.higgins@imperial.ac.uk

Sci. Adv. **9**, eadh3273 (2023)
DOI: 10.1126/sciadv.adh3273

The PDF file includes:

Figs. S1 to S12
Tables S1 and S2
Legends for data S1 and S2
Legend for movie S1

Other Supplementary Material for this manuscript includes the following:

Data S1 and S2
Movie S1

Figures

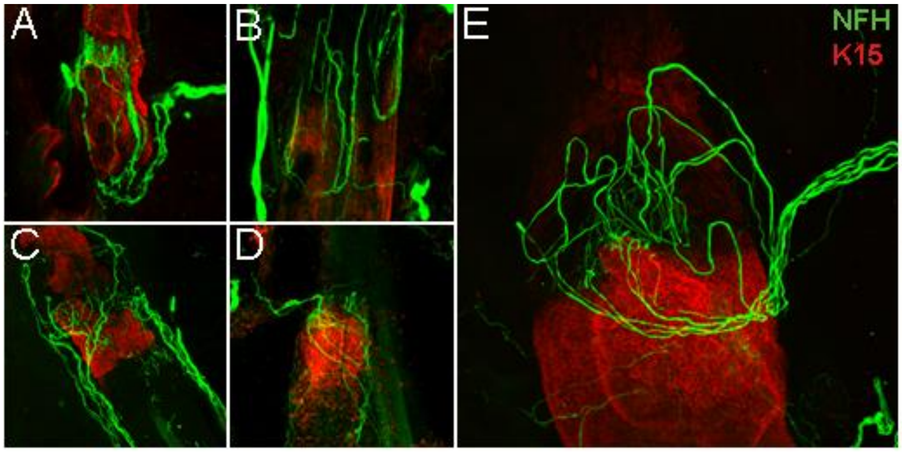

Figure S1. Volumetric imaging of human hair follicles from 7 individuals. (A) Longitudinal and circumferential LTMRs around the K15 positive hair follicle cells. (B-D) Longitudinal endings stained above the K15 positive hair follicle cells. (E) Longitudinal and circumferential endings above the K15 positive cells. All panels stained with antibodies against NFH (green) and K15 (red). Scale bars A-D: 500µm, Scale bar E: 200µm

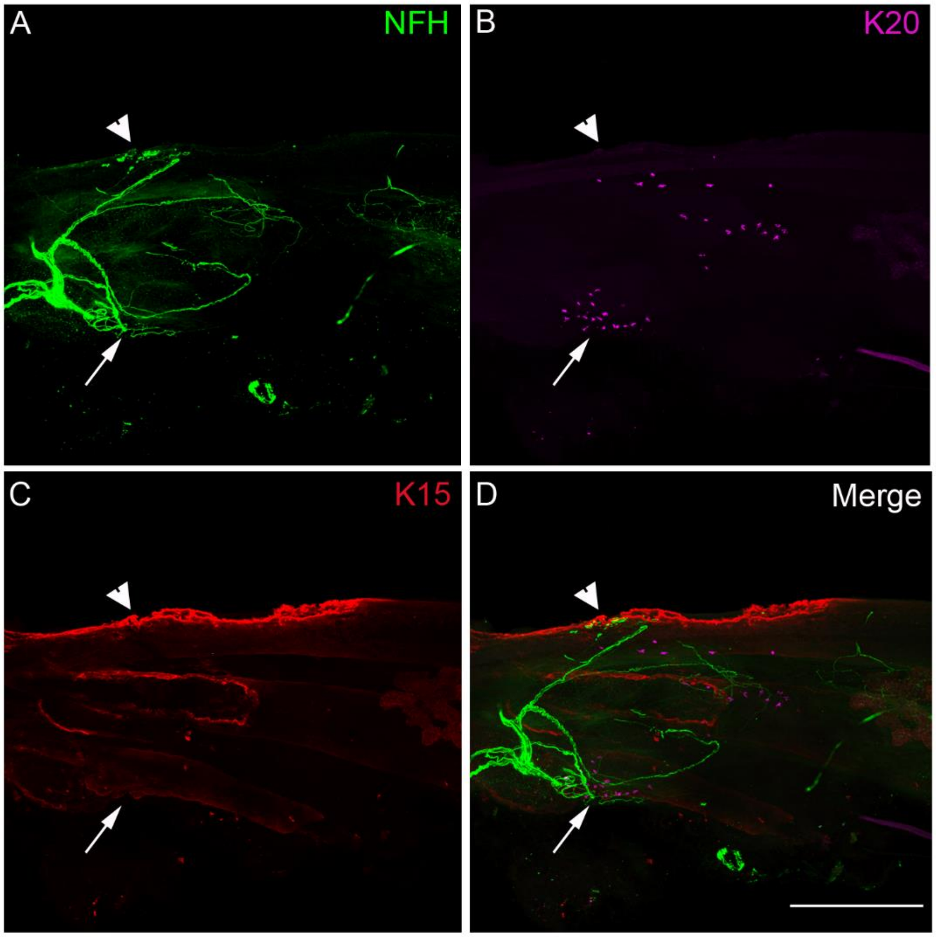

Figure S2. Confocal imaging of a follicular unit. (A) NFH positive neuronal nerve endings around hair follicles in a follicular unit. (B) K20 positive merkel cells located within the outer root sheath of hair follicles. (C) K15 marks 3 separate hair follicles in this follicular unit. (D) Merged image showing regions of the follicle with innervation, but no merkel cells (arrowhead) within one follicle, then a densely innervated area with merkel cells (arrow) within another follicle. Scale bar: 200μm

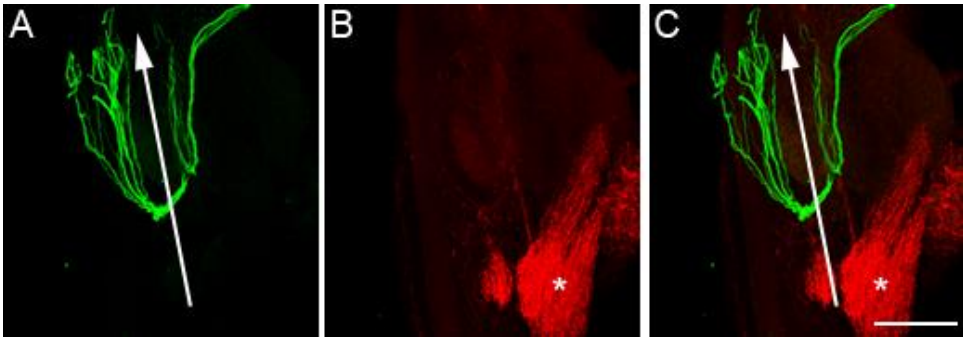

Figure S3. TH and NFH imaging in a human hair follicle. (A) NFH staining around the follicle (arrow running along length of follicle and pointing to skin surface). (B) TH staining of the sympathetic nerve in the APM (asterix). (C) Overlaid image showing LTMR localisation above the entry point of the APM. Scale bar. 100μm

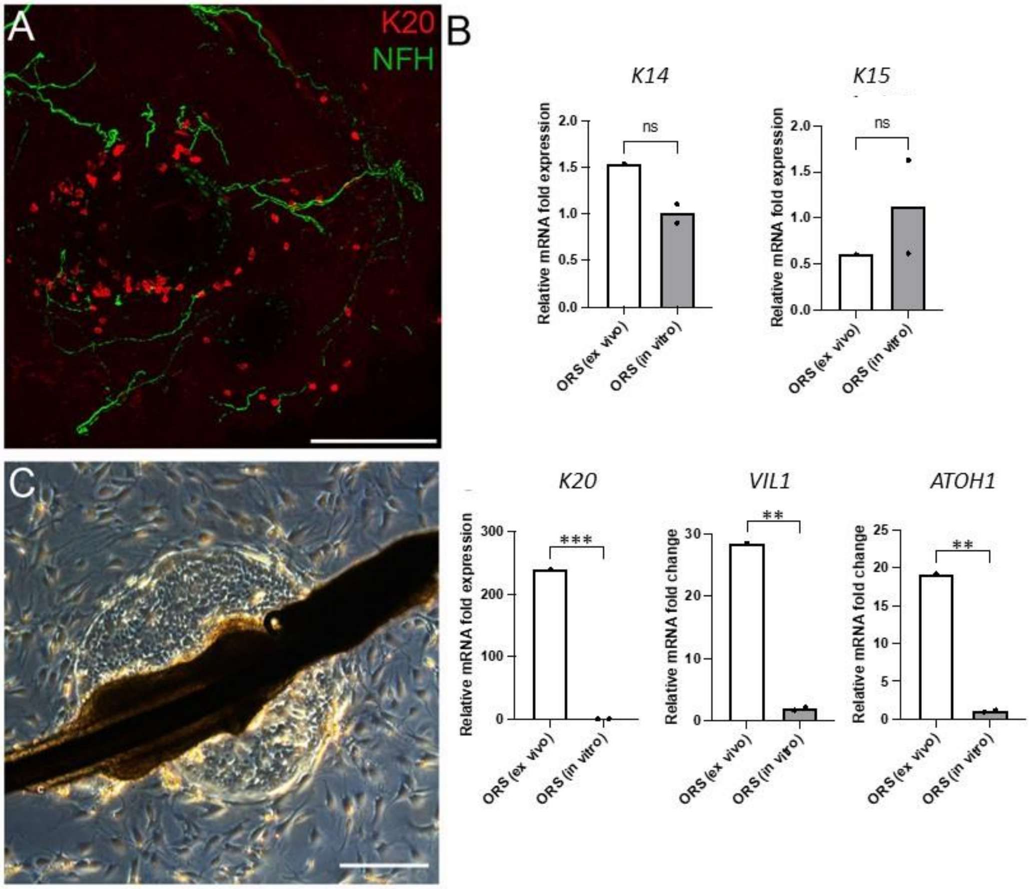

Figure S4. Merkel cells in the human hair follicle. (A) K20+ Merkel cells are located within the ORS of human hair follicles. (B) Gene expression of Merkel cell markers (*K20*, *VIL1* and *ATOH1*) is seen in plucked hair fibres (ORS ex vivo), while this expression is lost during culture (ORS in vitro). (C) Example image of an explant culture of ORS cells. Scale bars. 100μm

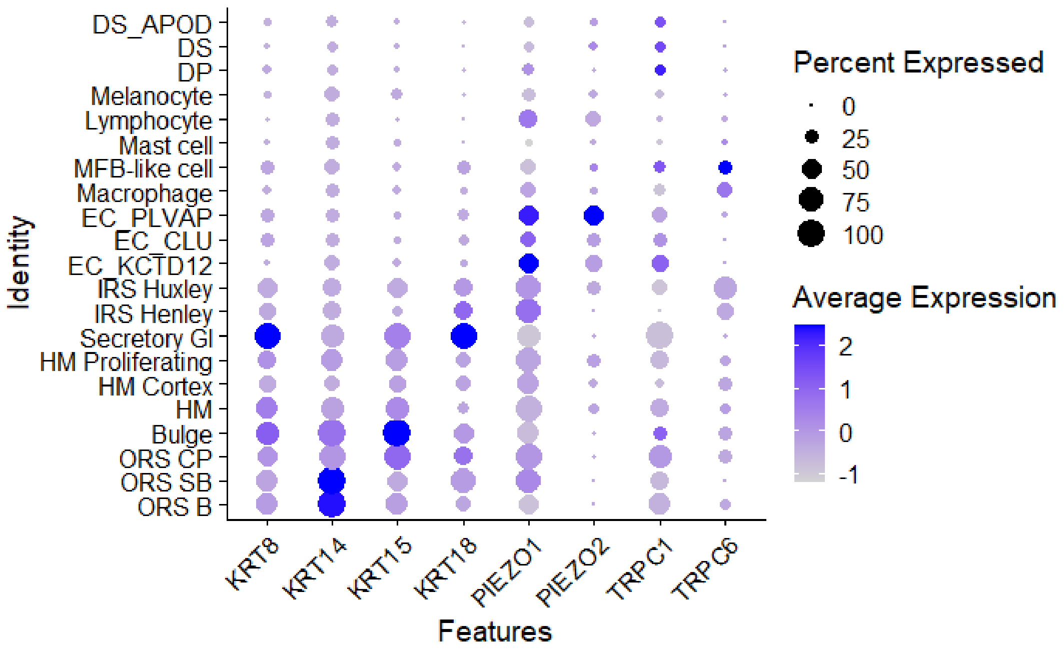

Figure S5 – scRNAseq data in human hair follicles. RNA expression of *KRT8*, *KRT14*, *KRT15*, *KRT18*, *PIEZO1*, *PIEZO2*, *TRPC1*, and *TRPC6* (Features) in anagen human hair follicles GSE193269 across cell clusters representative of various hair follicle compartments (Identity). Size of dots represents the total percentage of cells within that cell identity differentially expressing genes of interest. Colour of dots instead represent the average expression level within that cluster.

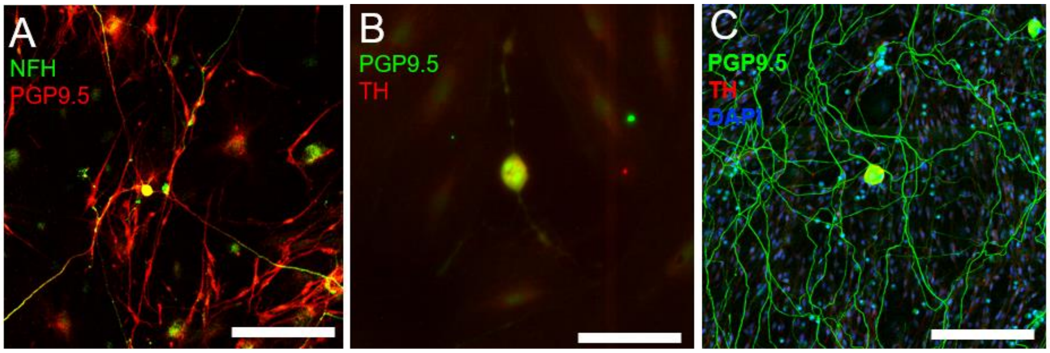

Figure S6. Characterization of neurons in DRG and ORS-DRG cultures. (A) Immunostaining of DRG cultures show LTMRs expressing NFH and PGP9.5. (B) TH+ C-LTMRs were also observed in the co-culture system with ORS cells. (C) Neurons PGP9.5 and Th+ show highly developed axonal network with ORS cells (DAPI). Scale bars: A = 100µm, B = 15µm, C = 50µm.

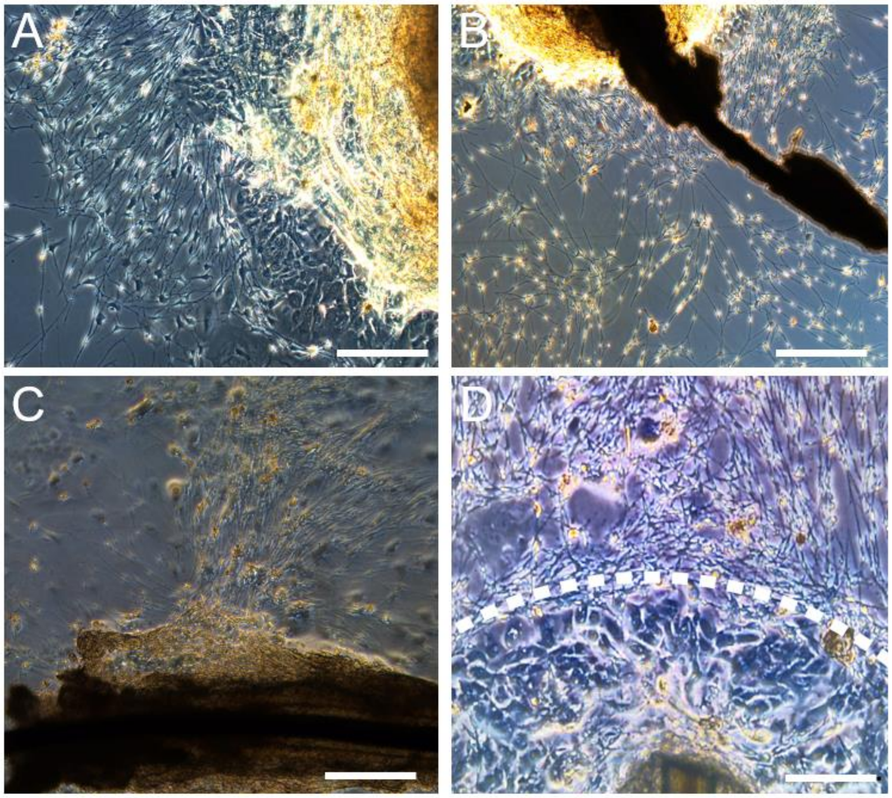

Figure S7. Stereomicroscope images of ORS-DRG co-cultures. (A-C) Examples of co-cultures between murine DRG neurons and human ORS cells. (D) Example of ORS cells and DRG neurons showing connectivity between the ORS cells region (dashed line) and the DRG neurons. Scale bars: A, B, C = 80µm, D = 50µm.

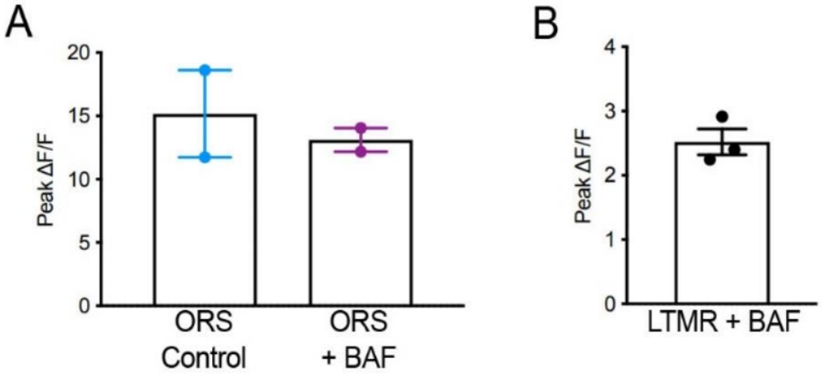

Figure S8. Effect of Bafilomycin on calcium transients in vitro. A) Calcium peaks in 2 ORS cells with and without Bafilomycin (BAF) after tactile stimulation of ORS cells. B) Calcium peaks in 3 LTMRs with BAF, after direct stimulation of LTMR (n=3).

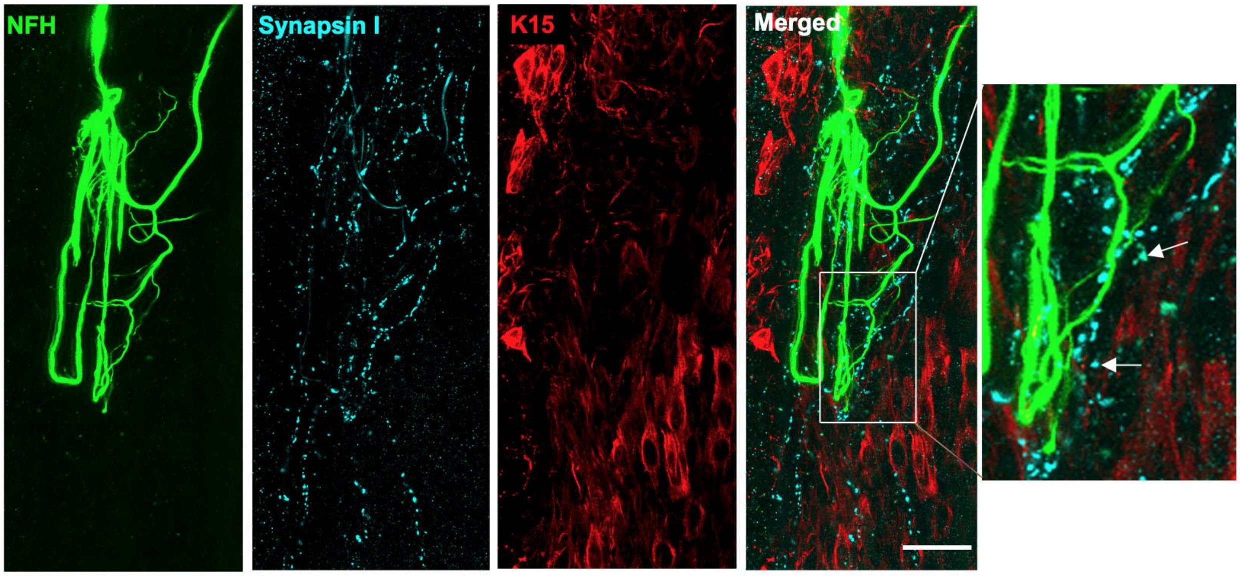

Figure S9: Synaptic vesicles in vivo. Immunofluorescence of NFH, Synapsin 1 and K15 in human hair follicle showing synaptic vehicles (arrows in inset) between the K15+ follicle cells and NFH+ nerve.

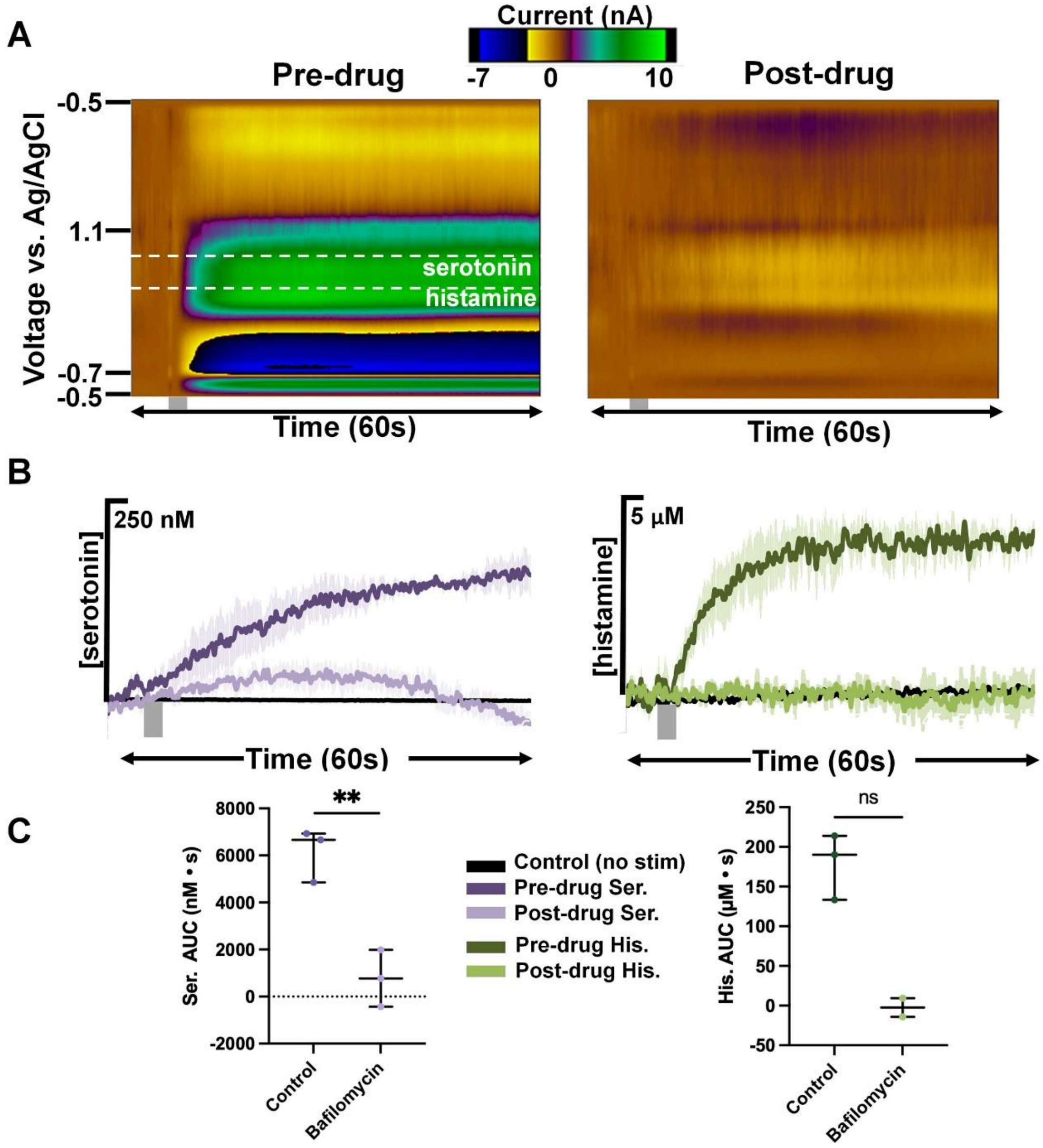

Figure S10: Direct stimulation of ORS with and without bafilomycin. FSCV analysis of neurochemical release in ORS cells in response to Bafilomycin (A) Representative colour plots of stimulated release in ORS cells before (left) and after (right) a 10 µM dose of bafilomycin for 1 hr. Gray bar denotes the time and duration of the stimulation. (B) Average traces of stimulated release of histamine (right, green) and serotonin (left, purple) in the ORS cells from 1 stim before (dark) and after (light) 10 µM bafilomycin. (C) Box and whisker plots comparing the area under the current vs. time curves of histamine (right, green) and serotonin (left, purple) release from the ORS cells before and after 10 µM bafilomycin.n = 6 stimulations (serotonin, pre and post bafilomycin) and n=5 stimulations (histamine, pre and post bafilomycin) in 1 culture from 1 patient for ORS cells.

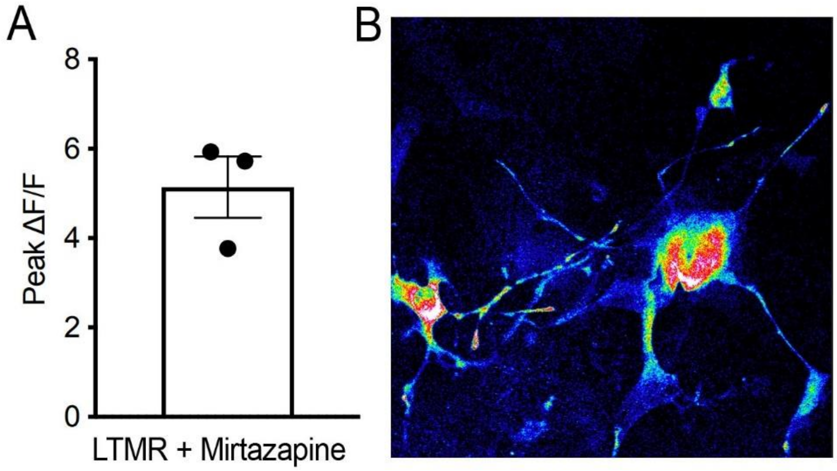

Figure S11: Effect of Mitazapine on calcium transients in DRG neurons in vitro. A) Direct stimulation of LTMR and resultant calcium peak in cells cultured with mirtazapine (n=3). B) Representative calcium transient image in a neuron in vitro after mechanical stimulation.

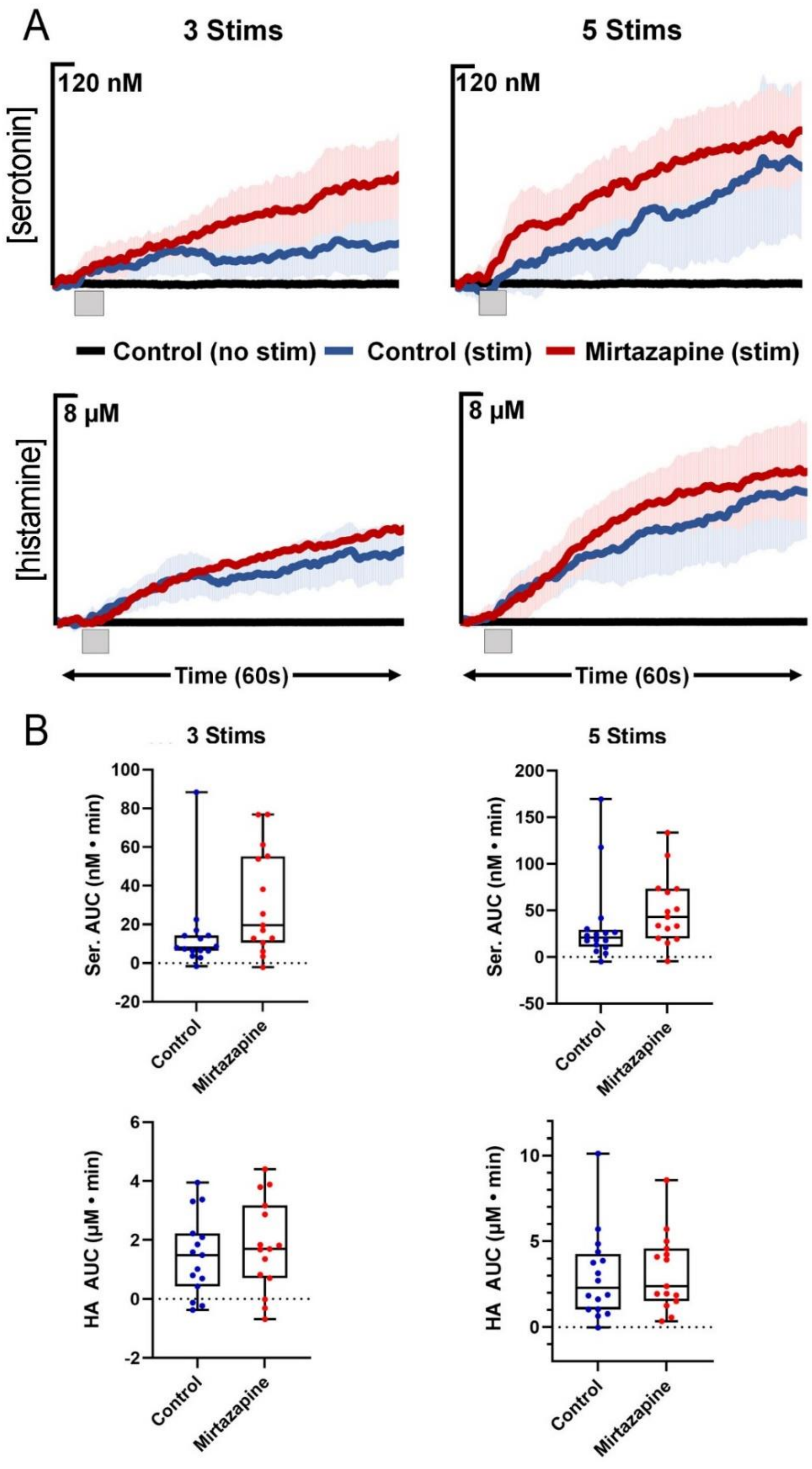

Figure S12: Direct stimulation of ORS with and without mirtazapine. (A) Average traces of stimulated release of serotonin (top) and histamine (bottom) from ORS cells with 3 or 5 Stim. Error bars are SEM. (B) Box and whisker plots comparing the area under the current vs. time curves of serotonin (top) and histamine (bottom) release from the ORS cells from 3 or 5 Stim. n = 15 stimulations in 5 ORS cultures from 4 patients.

Tables

Figure	Statistical test	Comparison	p-value
2F	One-way ANOVA, Bonferroni post hoc	Control vs No external Ca	0.0133
		Control vs ER Ca^{2+} depleted	0.3723
		No external Ca vs ER Ca^{2+} depleted	0.0003
2I	Unpaired t-test	ORS +/- GsMTx-4	<0.0001
3F	Unpaired t-test	LTMR +/- bafilomycin	<0.0001
3I	Unpaired t-test	LTMR +/- Apyrase	<0.0001
4G	Two-way ANOVA, Tukey-Kramer post-hoc	Histamine ORS vs KC 1 Stim	0.1320
		Histamine ORS vs KC 3 Stim	0.4780
		Histamine ORS vs KC 5 Stim	0.8720
4H	Two-way ANOVA, Tukey-Kramer post-hoc	Serotonin ORS vs KC 1 Stim	<0.0001
		Serotonin ORS vs KC 3 Stim	0.0042
		Serotonin ORS vs KC 5 Stim	0.0012
5C	Unpaired t-test	ORS +/- mirtazapine	0.9676
5F	Unpaired t-test	LTMR +/- mirtazapine	<0.0001
5H	Two-way ANOVA, Tukey-Kramer post-hoc	Serotonin -1 Stim +/- mirtazapine	0.0100
5I	Two-way ANOVA, Tukey-Kramer post-hoc	Histamine 1 Stim +/- mirtazapine	0.1740
S4	Unpaired t-test	*K14* ORS ex vivo vs in vitro	0.2000
		K15 ORS ex vivo vs in vitro	0.6602
		K20 ORS ex vivo vs in vitro	0.0004
		VIL1 ORS ex vivo vs in vitro	0.0093
		ATOH1 ORS ex vivo vs in vitro	0.0071
S8	Unpaired t-test	ORS +/- bafilomycin	0.6231
S10C	Two-way ANOVA, Tukey-Kramer post-hoc	Serotonin -1 Stim +/- bafilomycin	0.0048
	Two-way ANOVA, Tukey-Kramer post-hoc	Histamine 1 Stim +/- bafilomycin	0.0504
S12B	Two-way ANOVA, Tukey-Kramer post-hoc	Serotonin 3 Stim +/- mirtazapine	0.0707
		Serotonin 5 Stim +/- mirtazapine	0.2840
		Histamine 3 Stim +/- mirtazapine	0.5441
		Histamine 5 Stim +/- mirtazapine	0.7860

Table S1. Summary of p-values and statistical tests used for all graphical plots in manuscript.

**Gene**	**Forward Primer**	**Reverse Primer**	**Product length (bp)**
GAPDH	CGGGAAGCTTGTCATCAATGG	ATGACGAACATGGGGGCATC	206
K14	TCCAGGAGATGATTGGCAGC	GCACATCCATGACCTTGGTG	248
K15	AGATCGCTACTTACCGCAGCC	CCTGTCCATCCACTGACTCTTC	138
K20	TAAATGACCGTCTAGCGAGC	GCAGGACACACCGAGCATTT	200
VIL1	GCTGCTCTACACCTACCTCATC	TTCTGGTCCAGGATGACGGCTT	122
ATOH1	ACTTTGCAGGCGAGAGAGCA	CAGCTCCGGGGAATGTAGCA	143

Table S2. Primers used for real time PCR

Data S1. (separate file)

Calcium imaging data in ORS cells, ORS-LTMR co-cultures and ORS with drug treatments

Data S2. (separate file)

FSCV data in ORS cells and keratinocytes, and in ORS cells with drug treatments

Video S1. (separate file)

Timelapse video of example calcium imaging in ORS-LTMR coculture
